# Social Media Use and Mental Health Outcomes in Secondary School Students: A Systematic Review

**DOI:** 10.1192/bjo.2026.11117

**Published:** 2026-06-30

**Authors:** Mallika Punukollu, Saraswati Unnam, Padma Unnam

**Affiliations:** 1 NHS Greater Glasgow and Clyde, Glasgow, United Kingdom; 2 Wellington school, Ayr, United Kingdom

## Abstract

**Aims:**

Social media use is nearly universal among adolescents, yet its relationship with mental health remains contested. Much public and policy debate focuses on total time spent on social media, despite emerging evidence that qualitative aspects of use may be more relevant. Clarifying which dimensions of social media use are most strongly associated with mental health outcomes is essential for informing school, clinical, and policy interventions.

This review aimed to synthesise evidence on the association between social media use and mental health outcomes in secondary school students (approximately ages 11–18). We hypothesised that (1) problematic social media use would show stronger and more consistent associations with adverse mental health outcomes than time-based measures, and (2) contextual and experiential patterns of use would be more informative than frequency or duration alone.

**Methods:**

A rapid systematic review was conducted using a structured web-based search strategy to identify peer-reviewed systematic reviews, meta-analyses, and high-quality longitudinal or quasi-experimental studies examining social media use and mental health outcomes in adolescents attending secondary school. Eligible outcomes included depressive symptoms, anxiety symptoms, psychological distress, wellbeing, sleep disturbance, and self-harm-related outcomes. Social media exposure measures were categorised as time/frequency of use, problematic social media use (defined as compulsive or impairment-associated use), and patterns or experiences of use (e.g. active versus passive engagement, social comparison, feedback sensitivity). Findings were synthesised narratively by exposure category and outcome domain.

**Results:**

Across included evidence syntheses and longitudinal studies, associations between time spent on social media and mental health outcomes were generally small and inconsistent, with substantial susceptibility to confounding and reverse causality. In contrast, problematic social media use demonstrated more robust and consistent associations with depressive symptoms, anxiety symptoms, and psychological distress. Evidence examining patterns and experiences of use suggested that mechanisms such as social comparison, sleepdisruption, and emotional reactivity to online feedback may partially explain observed associations. Evidence evaluating school-based restriction policies alone showed limited and mixed effects on mental health outcomes.

**Conclusion:**

Findings support the hypothesis that problematic and impairment-related social media use, rather than total time spent, is most consistently associated with poorer mental health in secondary school students. Interventions and assessments should prioritise loss of control, sleep disruption, and emotional impact of use rather than focusing solely on duration. Stronger causal inference will require future studies using objective exposure measures and experimental designs.

